# Malocclusion and Pyorrhea Alveolaris

**Published:** 1919-03

**Authors:** 


### MALOCCLUSION AND PYORRHEA ALVEOLARIS

During the past year several articles have appeared dealing with the relation which exists between malocclusion of the teeth and inflammation of the gingival tissues. However, there is still much that can be said on the importance of these two subjects, and the correlation which exists between them. Almost every paper written by men interested in the treatment of pathologic conditions around the necks of the teeth, calls attention to the fact that malocclusion is an important etiologic factor, and many men also claim that the treatment of malocclusion with improperly fitted appliances produces inflammation of the gingival tissues. We are forced to admit both views are more or less correct.

Our attention was recently called to the relation of pyorrhea alveolaris and malocclusion by an article written by Dr. Thompson, published in the *Journal Allied Dental Societies.* Quoting the article, we find: ''Pyorrhea alveolaris, that term which covers a multitude of sins, finds its incipient cause in malocclusion, want of occlusion, bands irritating the pericementum, poorly filled roots, etc.'' Out of four cases given for the production of pyorrhea, three of them are intimately concerned with orthodontia; namely, malocclusion, want of occlusion, bands irritating the pericementum.

It seems to be a very well-established fact that the teeth which occupy malpositions can not be kept clean, and consequently lend themselves as etiologic factors in the production of gingival irritations. It is also known that bands irritating the peridental membrane are a predisposing, or an important, factor in the production of diseased gingival tissues; but, of course, every man claims that it is the other's bands which produce that harm. Therefore, while orthodontia is a strong factor in the prevention of gingival irritations, by the correction of malocclusion, it also in some instances becomes a prominent cause in the production of gingival irritations by the use of improperly constructed appliances, the principal one of which is improperly made bands.

A few years ago it was a common plan to use bands which were produced by manufacturers, and the band was sold with the understanding that it must be placed upon the tooth in the same form as it was made by the manufacturer. As a result of this, bands which were entirely too wide were used, and produced a great amount of harm owing to the fact that they were improperly fitted and improperly contoured. Certain bands were placed on the market which were made universally, and some were contoured to fit an upper right or a lower left molar. Manufacturers who possessed no knowledge of the anatomy of the teeth and who were able to get dentists to use such bands, necessarily unknowingly became factors in producing gingival irritations, because no clamp band is made that will fit an upper molar that can be used on a lower molar unless it is entirely recontoured and reshaped.

We also find men who advocate the making of bands very wide, thereby avoiding any possibility of their coming off the tooth during orthodontic treatment. There is no other factor that is producing so much gingival irritation as the wide bands made with the sole purpose of being cemented so that they will never become loosened. We believe that it would be more hygienic and much less harmful to use a band which is narrow, even though it would become loosened occasionally by the stress of mastication, than to use a wide band jammed down upon the gingival tissues to such an extent as to produce strangulation necrosis, along with the resulting irritation and infection which follows. Therefore, realizing the fact that orthodontia in correcting malocclusion, is a large factor in preventing gingival irritations, we must also look at the other side of the question and realize that the improperly constructed appliance will produce just as much harm as teeth which occupy malposition. However, with the properly constructed regulating appliance made with the knowledge of the shape of the tooth and the physiology of the soft tissue, orthodontia becomes one of the greatest hygienic measures in preventing the inflammation of the gingival tissue that we have.—Editorial in *International Journal of Orthodontia.*

